# Academic Well-Being in Higher Education: A Cross-Country Analysis of the Relationship Between Perceptions of Instruction and Academic Well-Being

**DOI:** 10.3389/fpsyg.2021.766307

**Published:** 2021-12-03

**Authors:** Dana K. Donohue, Juan Bornman

**Affiliations:** ^1^Northern Arizona University, Flagstaff, AZ, United States; ^2^University of Pretoria, Pretoria, South Africa

**Keywords:** academic well-being, power distance, quality of instruction, income, cultural values

## Abstract

The purpose of this research was to explore the relationship between university students’ perceptions of the overall quality of instruction (PQI) they experienced since COVID-19 and their academic well-being. This relationship was examined in the context of a moderated moderation with students’ household income and the cultural value of power distance (PD), which measures the extent to which less powerful members of an organization expect and accept that power is unequally distributed. Two countries with societally moderate levels of PD (South Africa and the United States) were assessed. Moderated moderations between PQI, income, and PD were found for the academic well-being of students from both the United States and South Africa. The patterns of interactions were in some ways similar and other ways different, highlighting the complexity of how students may react to potential stressors in their academic environment. Potential explanations and implications of these results are discussed.

## Introduction

In recent years, there has been a growing emphasis on student well-being as an important educational outcome (Govorova et al., 2020). Since COVID-19, well-being has become even more of a concern due to the academic, financial, and health stressors that arose during the pandemic. Well-being has been described as a dynamic state encompassing the potential to achieve one’s personal and social goals (Borgonovi and Pál, 2016) and is associated with students’ academic performance across countries (Govorova et al., 2020). Relatedly, academic well-being encompasses factors that contribute to doing well academically, such as academic achievement, academic stress, and academic satisfaction (Shek and Chai, 2020). General and academic well-being have been associated with positive outcomes for students (Robinson and Snipes, 2009; Korhonen et al., 2014; Shek and Chai, 2020), and both may have been negatively affected due to the changes and challenges to university life during COVID-19.

One considerable change to university life was a rapid transition from in-person to online learning modalities; research with students in higher education worldwide found that 87% experienced such a transition (Aristovnik et al., 2020). This often resulted in alterations to course materials and the type of instruction students received (e.g., from in-person instruction to a more remote and/or asynchronous instruction). Students have reported varying degrees of dissatisfaction about these changes (Aristovnik et al., 2020; Means and Neisler, 2020; Qazi et al., 2020). The purpose of this research was to explore the relationship between university students’ perceptions of the overall quality of instruction (PQI) they experienced since COVID-19 and their academic well-being. Two interacting moderators were assessed within the context of this relationship: household income and the cultural value of power distance (PD). These moderators were chosen as they represent two important constructs that often influence students’ academic experiences in educational settings. Two countries with societally moderate levels of PD (South Africa and the United States) were assessed.

### Academic Well-Being

In many fields, it is now essential to earn a university degree to practice professionally (e.g., health, law, engineering, education). A university degree is associated with a variety of positive outcomes including better health and healthcare, more employment security, and a higher salary when compared to people without degrees (Ma et al., 2016). For these reasons, it is important that students persist in their studies until they graduate. One precursor to students’ degree persistence is their academic well-being, which includes thoughts and behaviors that contribute to doing well in school, like achievement, academic satisfaction, and stress (Shek and Chai, 2020). In diverse samples, high academic well-being has been associated with lower rates of dropout (Korhonen et al., 2014), and with hope, optimism, self-efficacy (Robinson and Snipes, 2009), as well as with positive youth development (Shek and Chai, 2020).

COVID-19 may have negatively impacted students’ academic well-being. Because of the global pandemic and subsequent social distancing, many students were no longer permitted to attend class in person, were required to move out of dorms, lost jobs, and experienced anxiety about their own and loved ones’ health. In addition to the social and financial losses experienced, students may also have experienced “academic loss” because of the expeditious changes to their courses as online learning became a necessity rather than a choice. If students wanted to continue their studies, they had to take their courses online (Aristovnik et al., 2020).

Online learning is a format with which students are often ambivalent. On the one hand, online classes offer flexibility and can be more accessible to those with work and family responsibilities or who live in remote locations (Murphy, 2020); conversely, online courses require independent learning which can make engaging with the content more challenging, can eliminate the sense of community in the classroom (Song et al., 2004), and can subsequently lead to higher rates of course withdrawal (Frydenberg, 2007). Taken together, there are many potential issues that can arise when taking online courses in higher education; consequently, students’ academic well-being may have been negatively impacted by the worldwide transition to this learning modality during COVID-19.

### Quality of Instruction

Sogunro (2017) stated that quality of instruction was the “raison d’être” university students provided as a source of motivation in their studies. One motivator is the passion that instructors feel for topics, which can be contagious, with students likewise developing passion for the topics they are learning (Schunk et al., 2008). Another reason is that knowledgeable instructors choose and create appropriate materials to promote discussions, critical thinking, and learning. A third reason is that effective, high-quality instruction involves the dynamic delivery of the content (Sogunro, 2017), which can create an engaging learning environment.

Arguably, all instructors want to provide high-quality instruction. However, the quick transition to online courses due to COVID-19 created a unique situation that left many instructors unprepared, sometimes with little assistance for course design and technical support, and little time to ensure that altered course materials were of high quality and met the learning needs of students (O’Keefe et al., 2020). Research with students in higher education found that, since the onset of the pandemic and its accompanying changes to classes, 31% of students in the United States (Means and Neisler, 2020) reported moderate to strong dissatisfaction with the quality of instruction and students within Africa reported the lowest overall satisfaction with their instruction since the pandemic, when compared to students on other continents (Aristovnik et al., 2020). In addition to dissatisfaction with instruction, students have reported increased stress, an inability to focus, with some reporting that their online classes were no longer their priority (Herold and Chen, 2021).

### Income

One variable that might moderate the relationship between students’ PQI and academic well-being is their income, particularly when considering the evidence that worldwide, university students from low-income backgrounds experienced significantly more challenges, both academic and personal, than their more affluent peers during the COVID-19 pandemic. In the United States, low-income students reported less access to the internet and were more likely to drop courses because of low grades that could result in being ineligible for future financial assistance for their classes (Rodríguez-Planas, 2020). Personal challenges included more childcare responsibility, greater probability for illness and stress, housing challenges, and job loss (Rodríguez-Planas, 2020). Even in pre-pandemic times, the academic challenges faced by low-income South African students were many and varied, including the costs of housing, food, books, student fees, transportation costs, and sometimes a pressure to use their bursaries to provide their families with financial assistance (Mngomezulu et al., 2017). During COVID-19, many South African households lacked internet access (as Wi-Fi is restricted and data costs are high), computers, and consistent electricity due to a relatively common occurrence of load shedding (Dube, 2020). Because of these myriad challenges, students from low-income households worldwide likely experienced more difficulties, both academic and social, during COVID-19 and this may have impacted their academic well-being.

### Power Distance

Culture is the “software of the mind” (Hofstede, 2001), including the collective beliefs and values of a group (Triandis, 1996) and has been suggested to be a vital moderator when understanding how people perceive and respond to their experiences (Li et al., 2019). Hofstede’s Cultural Dimensions Theory (Hofstede et al., 2010) is a paradigm that describes six cultural values (power distance, indulgence, masculinity, individualism, long-term orientation, uncertainty avoidance) and the degree to which various countries hold these values. In this research, the cultural value of power distance (PD) was the focus. PD involves the degree to which individuals, societies, and nations accept social inequality as a natural occurrence (Hofstede, 2001). In educational research, PD has been suggested to be a key cultural value because of the inherent status hierarchies between student and instructor (Cortina et al., 2017).

In societies that score high on PD, there is deference to those in high status positions, whereas low-scoring societies view leaders and their subordinates more equally. However, PD can vary between people within societies; therefore, societal scores should not be applied to individuals. For example, a review of the literature on PD and well-being concluded that while PD negatively predicted the subjective well-being of countries, it positively related to individual-level outcomes, like work satisfaction (Daniels and Greguras, 2014).

On the individual-level, PD has been found to moderate the relationship between income inequality and subjective well-being (Li et al., 2019). PD may interact with income because it affects how people view inequality situations; for example, in school settings, high PD students may expect and accept that instructors have more power in the course dynamic. Therefore, students who are low-income and high PD may be more tolerant of low PQI than those who are low-income and low PD, who are less accepting of power differentials. Acceptance of inequality may, in turn, associate with higher well-being.

Research has found that students with high PD values view their instructors as knowledgeable, higher status, and less approachable when compared to students with lower PD values (Hwang and Francesco, 2010). In an online learning setting, students with high PD values were found to expect a strong instructor presence on the discussion board to guide discussions and indicate whose posts were correct and whose were not (Zhang, 2013). Conversely, in educational institutions with lower PD values and more egalitarian relationships among students and instructors, students reported high feelings of belongingness (Cortina et al., 2017).

PD has been previously conceptualized as a moderating variable with the outcome of well-being. In research about workplace relationships between managers and their subordinates, PD was found to moderate the relationship between abusive supervision by managers and employee well-being, where high PD acted as a protective buffer against manager negativity (Lin et al., 2013). In the workplace context, those high in PD were accepting of the imbalance of power and therefore viewed the abusive supervision as irrelevant to their well-being (Lin et al., 2013). Other scholars have likewise indicated that PD is an important moderator in the relationship between unfairness and justice because those high in PD are more accepting of inequality (Daniels and Greguras, 2014).

In this research with a focus on the academic context, PD was conceptualized as a moderator of income, whereas income was conceptualized as a moderator in the relationship between PQI and academic well-being. It was expected that the relationship between students’ PQI and academic well-being would depend on income, which would then depend on PD.

### Purpose

Academic well-being is an important precursor to students’ persistence in earning their degrees but may have been negatively affected during COVID-19. The purpose of this research was to examine students’ PQI and its effect on well-being, which was hypothesized to be moderated by income, which itself was hypothesized to be dependent on PD. This is termed a “moderated moderation” but is more widely known as a three-way interaction (Hayes, 2018).

The premise is that students have different expectations for what their school experiences should be like; students who have their expectations met or exceeded will have higher academic well-being than those who do not have their expectations met. Receiving high-quality instruction is one important academic expectation that students hold; however, its effect on well-being could depend on income: students from low-income backgrounds might feel they are wasting limited resources on their classes due to low PQI, which would lead to even lower well-being than low PQI would for a student who has more resources. Moreover, the income differences in the link between PQI and well-being may be PD dependent; for example, the income differences that moderate the PQI–academic well-being link could be smaller among those with high PD than low PD because high PD can serve as a buffer for well-being in inequality situations.

With moderation analyses, interaction effects are first assessed. If a significant interaction is found, then the main effects should not be interpreted, as the effect of one variable depends on—or moderates—another. Because of this, the highest order three-way interaction was first hypothesized. If found to be statistically significant, then the remaining hypotheses for that country were disregarded because lower-order effects should not be interpreted. If the three-way interaction was found to be non-significant, then the two-way interactions would be analyzed for that country. Similarly, if a two-way interaction was found, then the main effects involving those variables should not be interpreted.

The specific hypotheses were as follows:

*H1*: The relationship between PQI and academic well-being will be moderated by income, which will be moderated by PD for students in the United States (H1a) and South Africa (H1b).

*H2*: If H1 is unsupported, there will be a two-way interaction, where:

- There will be a relationship between PQI and well-being, moderated by income for students in the United States (H2a) and South Africa (H2b).
- There will be a relationship between PQI and well-being, moderated by PD for students in the United States (H2c) and South Africa (H2d).
- There will be a relationship between income and well-being, moderated by PD for students in the United States (H2e) and South Africa (H2f).

*H3*: If H2 is unsupported, there will be main effects, where:

- There will be a main effect of PD on well-being for students in the United States (H3a) and South Africa (H3b).
- There will be a main effect of PQI on well-being for students in the United States (H3c) and South Africa (H3d).
- There will be a main effect of income on well-being for students in the United States (H3e) and South Africa (H3f).

## Materials and Methods

### Participants

#### United States

Data were collected at two universities in the southwestern United States. Undergraduate students enrolled in psychology classes had a range of research studies in which they could participate. If they chose to participate in this online study, they signed into the study and were brought to an online data collection program. There were n=896 participants whose data were used for the moderation analysis, but some were missing data on their demographics. The age data (total *n*=889) indicated participants ranged from 17 to 56 (*M*=19.01, *SD*=2.32) and reported their gender (total *n*=896) as *n*=153 males, *n*=731 females, *n*=5 transgender, *n*=5 “other,” and *n*=2 prefer not to disclose. Annual family income (total *n*=896) was reported to be $50,000USD or below by *n*=339 participants, and the remaining *n*=557 participants reported family incomes from $50,001USD and above.

#### South Africa

Data were collected from a large university in the Gauteng province. Permission from the Registrar was first granted to conduct the study. Following that, the Deans at five faculties were contacted to obtain permission to correspond with heads of individual departments. The heads of department were contacted, and they then shared the information letter about the research with a link to complete the survey with staff. Staff subsequently sent the link and information letter about the research to students.

A total of *n*=181 participants’ data were used for the moderation analyses, but some were missing data for demographics. For age (*n*=180), the data ranged from 18–42 (*M*=21.74, *SD*=3.54). Gender (*n*=181) was reported as *n*=43 males, *n*=135 females, *n*=1 transgender, and *n*=2 reported “other.” Annual family income (*n*=181) was reported to be around R705,500 (approximately $50,000USD) or below for *n*=102 participants and the remaining *n*=79 participants reported annual family income of R705,501 (approximately $50,001USD) or above.

### Materials

#### CVScale

The CVScale is a measure of Hofstede’s Cultural Value Theory (Hofstede et al., 2010) on the level of the individual rather than the level of society (Yoo et al., 2011). The Power Distance (PD) subscale was used for this research. It utilizes 5 items, such as, “People in higher positions should avoid social interaction with people in lower positions” that were measured on a 5-point Likert scale, ranging from 1=strongly disagree to 5=strongly agree. Higher scores indicated more PD. For the PD subscale, the alpha coefficients were *α*=0.86 for United States and *α*=0.84 for South Africa.

#### Perceptions of Academic Stress Scale

To measure academic well-being, the first 5 items on the Perceptions of Academic Stress Scale (PASS) were used (Bedewy and Gabriel, 2015). The PASS is an 18-item measure that uses a Likert scale from 1 to 5 (1=strongly disagree; 5=strongly agree). The first 5 items on the scale are positively worded and ask questions like, “I am confident that I will be a successful student” and “I make academic decisions easily.” Confirmatory factor analyses revealed marginal fit [CFI=0.897, RMSEA=0.119, *χ*^2^(10)=166.090, *p*<0.01]. Dropping the 5th item significantly improved model fit [CFI=0.959, RMSEA=0.110, *χ*^2^(4)=57.759, *p*<0.01] and so the first 4 items were used as a measure of academic well-being. Reliability estimates were United States *α*=0.76 and South Africa *α*=0.68.

#### Perceived Quality of Instruction

PQI since COVID-19 was assessed with one item measured on a 7-point Likert scale (1=extremely low to 7=extremely high). The question asked: “How would you rate the overall quality of instruction in your classes since COVID-19?” As can be seen in Table 1, the means for both the United States and South Africa are around the midpoint of the scale, which is similar to what other recent research has found on student course satisfaction since the pandemic (e.g., Means and Neisler, 2020).

**Table 1:** Mean, standard deviation, and range for summary scores between countries.

Variable	Country	Mean	SD	Range
Academic Well-being Summary Score	United States (*n*=896)	2.13	0.76	1–5
	South Africa (*n*=181)	2.11	0.72	1–4.25
Power Distance Summary Score	United States (*n*=896)	1.85	0.91	1–5
	South Africa (*n*=181)	1.89	0.93	1–5
Quality of Classes Score	United States (*n*=896)	4.29	1.45	1–7
	South Africa (*n*=181)	4.77	1.49	1–7
Income	United States (*n*=896)	5.02	3.01	1–9
	South Africa (*n*=181)	3.54	2.54	1–9

#### Annual Income

To measure income, participants were asked, “What is your annual household income? Please report your parental income if you are a dependent.” Participants were provided options from 1 (lowest income category) to 9 (highest income category). In the United States, the categories ranged from 1: $30,000USD and below to 9: $100,000USD and above. In South Africa, the categories ranged from 1: R423,300 (app. $30,000USD) and below to 9: R1,410,001 (app. $100,000USD) and above.

### Procedure

The IRB at the principal investigator’s home university approved the research and it was then approved by the other participating universities’ ethics boards. The data were collected from May 2020–December 2020 using an online data collection tool. If students agreed to participate, they clicked a link and were redirected to the anonymous survey where they were first provided with an informed consent that explained the purpose of the study, participation was voluntary, and they could withdraw from the study at any time without penalty.

## Data Analysis

SPSS and Amos Version 27 were used to analyze the data. The data were first analyzed for missing values and whether the patterns of missing data were completely at random. Little’s (1988) MCAR analyses indicated that the missing data were completely at random in the United States *χ*^2^(3)=2.13, *p*=0.55 and in South Africa, *χ*^2^(5)=3.77, *p*=0.58. Since the data were missing completely at random, the defaults for missing data were used: AMOS utilized Full Information Maximum Likelihood and SPSS PROCESS used listwise deletion.

Measurement invariance was analyzed using multi-group confirmatory factor analyses (CFA). These CFA analyses assess whether the scales are measuring the same phenomena across countries (Lee, 2018). Often the criteria for full measurement invariance are not reached, but partial measurement invariance can still be found by releasing the constraints on some item and/or intercept loadings. If the Chi-squared difference between the constrained and unconstrained scalar invariance models is not statistically significant, then group comparisons can be made (de Schoot et al., 2012). Full metric and scalar invariance were established for the Power Distance subscale and full metric and partial scalar invariance were established for the Perceptions of Academic Stress (PAS) subscale. Partial scalar invariance for the PAS was reached by un-constraining the intercepts for items 3 and 4 across groups (see Table 2 for model fit indices).

**Table 2:** Fit indices for models of measurement invariance.

	Overall models				Nested models (M2 compared to M1)				
Model	*χ*^2^(df)	*p*	CFI	RMSEA	Δ*χ*^2^(df)	Sig.	Δ*p*	ΔCFI	ΔRMSEA
**Power Distance Subscale**									
M1: Configural invariance	38.170(10)	0.00	0.988	0.051	–		–	–	–
M2: Metric invariance (Item 1 constrained to 1 across groups)	40.955(14)	0.00	0.989	0.042	2.786(4)	No	0.594	0.001	0.009
M3: Scalar invariance	48.005(19)	0.00	0.988	0.037	9.886(9)	No	0.360	0.000	0.014
**Perceptions of Academic Stress Scale (Items 1–4)**									
M1: Configural invariance	57.759(4)	0.00	0.959	0.110	–		–	–	–
M2: Metric invariance (Item 1 constrained to 1 across groups)	62.885(7)	0.00	0.957	0.085	5.127(3)	No	0.163	0.002	0.025
M3: Partial scalar invariance (Intercepts 3 and 4 unconstrained across groups)	68.008(9)	0.00	0.955	0.077	10.249(5)	No	0.068	0.004	0.033

**Figure 1:**
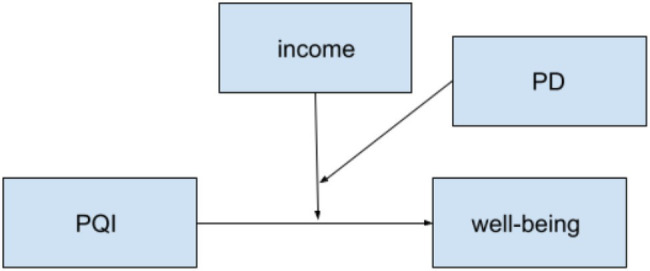
Hypothesized three-way moderation model for both countries.

**Figure 2:**
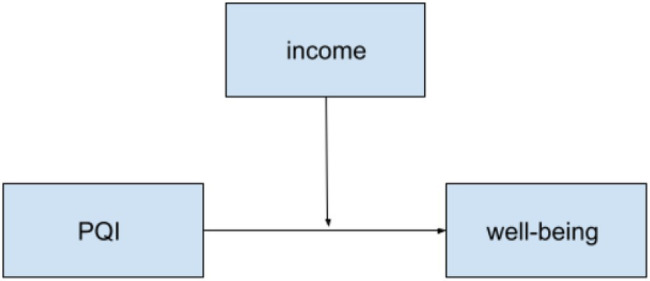
Hypothesized two-way moderation model between PQI and income on well-being for both countries.

**Figure 3:**
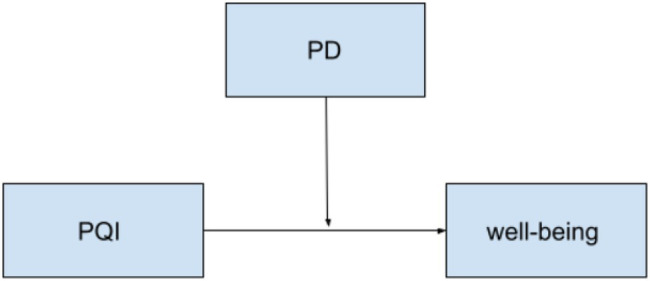
Hypothesized two-way moderation model between PQI and PD on well-being for both countries.

**Figure 4:**
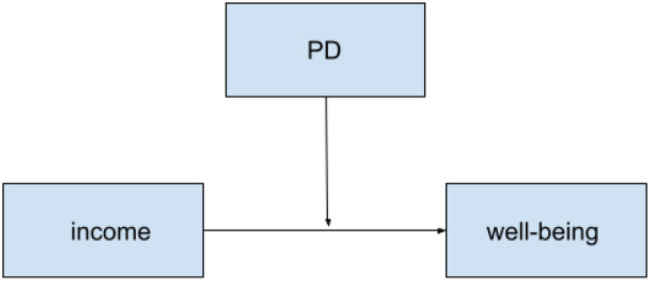
Hypothesized two-way moderation model between income and PD on well-being for both countries.

Next, the moderation hypotheses were assessed with the PROCESS macro-model 3 in SPSS, where there are two interacting moderators (Hayes, 2018). The items on the subscales for academic well-being and PD were averaged to create summary scores. The descriptive statistics by country for the measured variables can be found in Table 1 and the results of the moderation analyses are in Table 3. Figure 1 shows the hypothesized model to be tested for H1a and b, and Figure 2 illustrates the model for H2a and b, Figure 3 for H2c and d, and Figure 4 for H2e and f.

**Table 3:** Moderation analyses for quality of instruction, power distance, and income on students’ well-being.

Effect	*b*	*SE*	95% CI		*p*
**United States**	***R***^**2**^ **=0.15**		**LL**	**UL**	
Intercept	1.80	0.32	1.17	2.42	0.00
Quality	0.40	0.07	0.27	0.54	0.00
Income	0.22	0.06	0.10	0.34	0.00
Quality × Income	−0.04	0.01	−0.06	−0.01	0.00
Power distance (PD)	0.63	0.15	0.33	0.93	0.00
Quality × PD	−0.11	0.03	−0.18	−0.05	0.00
Income × PD	−0.11	0.03	−0.17	−0.05	0.00
Quality × Income × PD	0.02	0.01	0.01	0.03	0.00
**South Africa**	***R***^**2**^ **=0.10**		**LL**	**UL**	
Intercept	2.54	0.75	1.05	4.02	0.00
Quality	0.31	0.15	0.01	0.60	0.04
Income	0.46	0.18	0.10	0.82	0.01
Quality × Income	−0.10	0.04	−0.18	−0.03	0.01
Power distance (PD)	0.14	0.33	−0.52	0.80	0.68
Quality × PD	−0.05	0.07	−0.19	0.08	0.45
Income × PD	−0.15	0.09	−0.32	0.02	0.08
Quality × Income × PD	0.04	0.02	0.00	0.07	0.05

## Results

### United States

For students in the United States, a statistically significant three-way interaction was found between PQI, income, and PD on academic well-being (*b*=0.02, *SE*=0.01, *p*<0.01, 95% CI: 0.01–0.03; see Figure 2). This confirmed H1a. The unstandardized simple slope for income and PD, both one standard deviation below the mean, evinced a stronger effect (*b*=0.25, *SE*=0.03, *p*<0.00, 95%CI: 0.19–0.31), than the simple slope for students with low income and PD at the mean (*b*=0.19, SE=0.02, *p*<0.00, 95%CI: 0.14–0.23), and PD one standard deviation above the mean (*b*=0.12, *SE*=0.03, *p*<0.00, 95%CI: 0.06–0.18). The unstandardized simple slopes for moderate income and differing levels of PD were all relatively similar (*b*=0.18–0.20, all *p*<0.00). In contrast to low-income and middle-income students, the strongest effect for students with income one standard deviation above the mean was for high PD (*b*=0.23, *SE*=0.04, *p*<0.00, 95%CI: 0.15–0.30) and weakest for low PD (*b*=0.15, *SE*=0.03, *p*<0.00, 95%CI: 0.08–0.21).

### South Africa

For students in South Africa, a statistically significant three-way interaction was found between PQI, income, and PD on academic well-being (*b*=0.04, *SE*=0.02, *p*<0.05, 95% CI: 0.00–0.07; see Figure 3). This confirmed H1b. The unstandardized simple slopes for income and PD, for low-income students were all similar: one standard deviation below the mean for PD (*b*=0.18, *SE*=0.08, *p*<0.05, 95%CI: 0.04–0.34), the mean for PD (*b*=0.18, *SE*=0.05, *p*<0.00, 95%CI: 0.07–0.28), and one standard deviation above the mean for PD (*b*=0.16, *SE*=0.07, *p*<0.05, 95%CI: 0.03–0.29). The unstandardized simple slope for moderate income and low PD was non-significant (*b*=0.02, *SE*=0.05, *p*=0.62, 95%CI: −0.08–0.13), but was significant at moderate PD (*b*=0.09, *SE*=0.04, *p*<0.01, 95%CI: 0.02–0.16) and high PD (*b*=0.16, *SE*=0.05, *p*<0.01, 95%CI: 0.06–0.27). For high income students, the slope one standard deviation below the mean of PD was negative (*b*=−0.14, *SE*=0.07, *p*<0.00, 95%CI: −0.28–0.00) and was non-significant at the mean of PD (*b*=0.01, *SE*=0.05, *p*=0.80, 95%CI: −0.08–0.11), and one standard deviation above the mean of PD (*b*=0.17, *SE*=0.09, *p*=0.053, 95%CI: −0.00–0.34).

## Discussion

There were many negative consequences of the pandemic for some students in higher education. Considering the myriad stressors that students have experienced during the rapid transition to online courses, significant concern about their academic well-being arose. The purpose of this research was to examine how students’ PQI influenced their academic well-being and whether this relationship was moderated by students’ household income, which was itself moderated by their PD values. To this end, university students from two societally moderate PD countries (i.e., South Africa, United States) were assessed.

**Figure 5:**
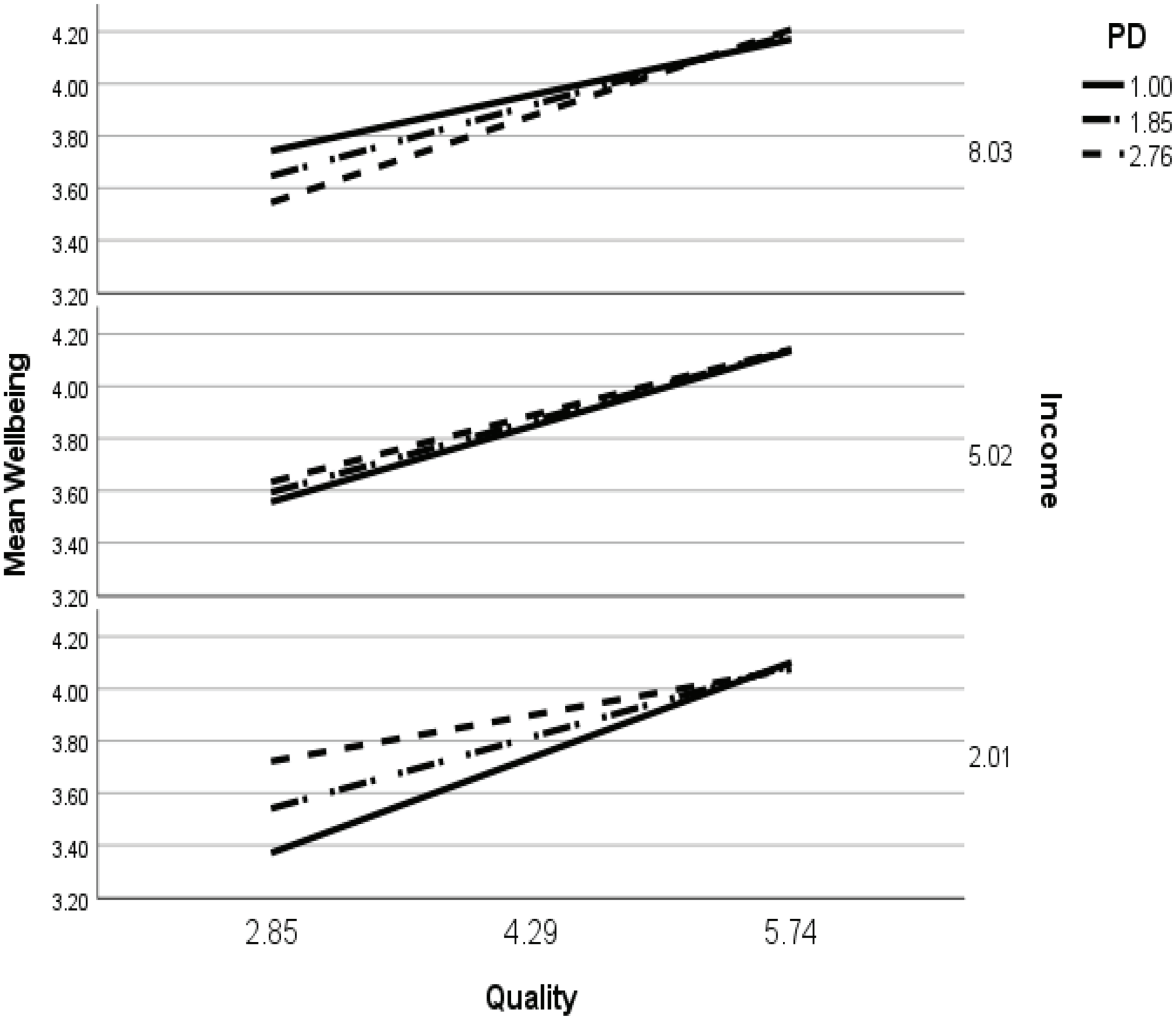
Simple slopes of PQI predict academic well-being for values of income and PD at 1 SD below the mean, the mean, and 1 SD above the mean for students from the United States.

**Figure 6:**
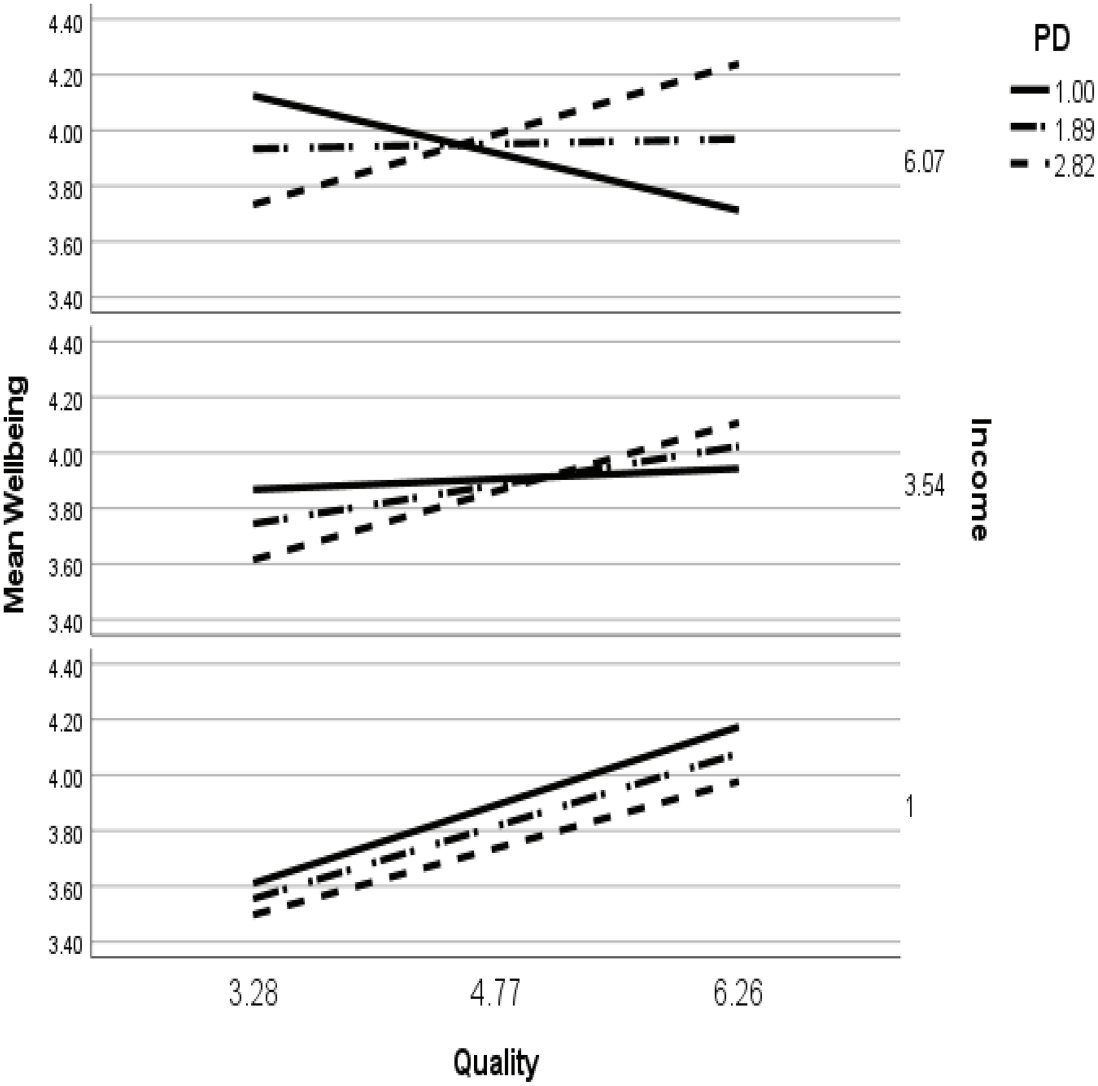
Simple slopes of PQI predicting academic well-being for values of income and PD at 1 SD below the mean, the mean, and 1 SD above the mean for South African students.

Three-way interactions between PQI, income, and PD were found for the academic well-being of students from both the United States and South Africa, confirming H1a and b. Illustrated in Figures 5, 6, distinct patterns of interactions for the students in these countries were evident. If perceptions of instruction were high, students from the United States exhibited equivalent academic well-being regardless of income and PD. When PQI was low, the results diverged for low- and high-income students. Students with low-income and high PD reported higher academic well-being than when low-income and low PD. As previously suggested (e.g., Lin et al., 2013; Daniels and Greguras, 2014; Li et al., 2019), high PD may act as a buffer against potentially unjust circumstances because people who adhere to beliefs about the need for social hierarchy are more likely to acquiesce to inequality situations, which associates with higher well-being. This may be particularly salient for students in the United States with low income, potentially with limited resources, and who perceive low PQI.

In contrast, when students were high-income and experienced low PQI, those with lower PD had higher academic well-being than those with higher PD. A similar finding was found for high-income, low PD South African students. Perhaps students with low PQI and high income were making downward social comparisons with their less economically fortunate peers, a phenomenon that has been found to enhance the well-being of people in the United States (Vogel et al., 2014) and middle-income countries (Antinyan, 2016). Seeing how their peers and instructors were struggling during the pandemic, they were more accepting of the low PQI and viewed it as irrelevant to their academic well-being.

Unlike students from the United States, the results diverged for high-income South African students when PQI was high: Students with higher PD had higher well-being than those with lower PD. It is reasonable to assume that high-income students with high PD had high expectations for PQI, and when it was met, these students reported high academic well-being. Why those low in PD would have such low well-being is more perplexing. It could be that high-income students who are low in PD, but high in PQI may have been particularly affected by the academic and social losses that were caused by the rapid transition to remote instruction during COVID-19. High-income South African students may be particularly accustomed to some of the perks of being at university because they experience fewer financial stressors than their less affluent peers. Though their instructors, who they viewed as relatively equal to them on the social hierarchy, were perceived as providing high quality instruction, this did not compensate for the numerous other academic and social losses that they experienced which negatively influenced their academic well-being.

In summary, the patterns of interactions for students from the United States and South Africa were in some ways similar and other ways different, highlighting the complexity of how students may react to stressors in their academic environment. Students from the United States reported relatively similar levels of academic well-being when PQI was high, regardless of their income and PD, emphasizing the importance of high-quality instruction for all groups of students in the United States Conversely, students from South Africa were found to have higher well-being when they had low PD, regardless of income when PQI was low, but low PD did not associate with academic well-being when PQI was high if students were middle- or high-income. This suggests that South African students’ well-being benefits from high PQI, but how this associates with their well-being can also depend on their PD.

### Implications

Students’ academic well-being is an important precursor in their persistence in higher education. Given this, there are several implications of this research. One implication is the importance of high quality of instruction for students’ well-being: with the exception of middle- and high-income South African students with low PD, the results consistently showed that high PQI was associated with higher academic well-being for students from the United States and South Africa, no matter their income or PD. Students’ perceived quality of instruction is a considerable motivator in their studies (Sogunro, 2017) and so ongoing “quality control” of classes is important when contemplating the ways that COVID-19 has impacted classes.

Another consideration is how financial resources and cultural values can interact to influence students’ academic well-being. Li et al. (2019, p. 1237–1238) suggest that “culture moderates how people feel about financial inequality in terms of their own finances, which then translates to how people feel generally…emphasizing the crucial role of culture as a moderator of how people react to a given reality”. Considering that students from low-income families are less likely to persist in their studies under ordinary circumstances (Africa, 2005), universities around the world should examine how the pandemic negatively impacted low-income students and engage in efforts—like quality, evidence-based instruction that serves low-income students—to mitigate the challenges they experienced during the pandemic, so they can persist in their studies.

A third implication is whether the cultural values that students hold can be malleable, and, if so, whether adopting certain values can promote their academic well-being. In their research about abusive managers and employee well-being, Lin et al. (2013) suggest that employees can *develop and hold* PD values to reduce the detrimental ways that abusive supervision affects them, thereby helping those employees maintain their well-being. Perhaps universities can help promote PD values that are associated with student success in their context. This could potentially be accomplished by developing a university climate that encourages appropriate PD values in the form of student attitudes toward their classes and/or in the form of authoritarian or egalitarian teaching styles by instructors. However, as the results of this research suggest, the way that PD and income interact to affect outcomes may differ by country and culture so there likely will not be a one-size-fits-all approach for universities.

### Limitations and Future Directions

There were some limitations in this research. First, these data were correlational and so cause-and-effect cannot be established; it could be that high academic well-being leads to high PQI, that high PQI leads to high well-being, or that both are related to an unmeasured third variable, like high achievement. Next, only a few universities were sampled and so the results of this research may not be generalizable to the entire population of students in higher education in South Africa or the United States. Another limitation is that students were only asked about the overall quality of instruction that they had received since COVID-19, which may have obfuscated important distinctions between their perceived instruction in different classes. The primarily female composition of the sample was another limitation, and the results may be different if more males participated. For these reasons, replication of these results is needed.

Future directions could capitalize on these limitations by conducting similar research in additional countries and universities, by allowing students to distinguish between their PQI in different courses, and by ensuring more gender diversity in their samples. Researchers could also try to analyze these relationships in a more experimental setting or follow students over time to better establish causality.

## Conclusion

Through high-quality instruction, students can become passionate about their studies and engage in learning behaviors that promote deep and critical thinking about the content which they are learning. Despite the many challenges that COVID-19 created in higher education, it is vital that instructors continue to provide students with classes that stimulate their learning, achievement, critical thinking, and that promote their academic well-being. Together, this can motivate students to continue in their degree persistence and flourish in their professions.

## Data Availability Statement

The raw data supporting the conclusions of this article will be made available by the authors, without undue reservation.

## Ethics Statement

The studies involving human participants were reviewed and approved by Northern Arizona University Institutional Review Board. The patients/participants provided their written informed consent to participate in this study.

## Author Contributions

DD wrote the manuscript and conducted the analyses. JB edited the manuscript and provided key insights for the discussion. All authors contributed to the article and approved the submitted version.

## Conflict of Interest

The authors declare that the research was conducted in the absence of any commercial or financial relationships that could be construed as a potential conflict of interest.

## Publisher’s Note

All claims expressed in this article are solely those of the authors and do not necessarily represent those of their affiliated organizations, or those of the publisher, the editors and the reviewers. Any product that may be evaluated in this article, or claim that may be made by its manufacturer, is not guaranteed or endorsed by the publisher.
